# Persistent High Burden of Advanced HIV Disease Among Patients Seeking Care in South Africa’s National HIV Program: Data From a Nationwide Laboratory Cohort

**DOI:** 10.1093/cid/ciy045

**Published:** 2018-03-04

**Authors:** Sergio Carmona, Jacob Bor, Cornelius Nattey, Brendan Maughan-Brown, Mhairi Maskew, Matthew P Fox, Deborah K Glencross, Nathan Ford, William B MacLeod

**Affiliations:** 1 Department of Molecular Medicine and Haematology, School of Pathology, Faculty of Health Sciences, University of the Witwatersrand, Johannesburg; 2 National Health Laboratory Service, Johannesburg; 3 Health Economics and Epidemiology Research Office, Department of Internal Medicine, School of Clinical Medicine, Faculty of Health Sciences, University of the Witwatersrand, Johannesburg, South Africa; 4 Department of Global Health, Boston University School of Public Health, Massachusetts; 5 Department of Epidemiology, Boston University School of Public Health, Massachusetts; 6 Southern Africa Labour and Development Research Unit, University of Cape Town, South Africa; 7 World Health Organization, HIV/AIDS, Geneva, Switzerland

**Keywords:** advanced HIV disease, CD4 cell count, morbidity, mortality, South Africa

## Abstract

**Background:**

The South African national HIV program has increased antiretroviral therapy (ART) coverage over the last decade, supported by policy changes allowing for earlier ART initiation. However, many patients still enter care with advanced (<200 cells/μL) and very advanced (<100 cells/μL) HIV disease. We assessed disease progression at entry to care using nationwide laboratory data.

**Methods:**

We constructed a national HIV cohort using laboratory records containing HIV RNA loads and CD4 counts from 2004 to 2016 to determine entry into care. We estimated numbers and proportions of adults with the first CD4 count <100 cells/ μL or 100–199 cells/μL. We calculated relative risks of presenting with advanced disease associated with male sex.

**Results:**

8.04 million first CD4 results were identified. From 2005 to 2011, the proportion of patients entering into care with CD4 count <200 cells/μL declined from 46.8% to 35.6%. From 2011 onward, the proportion of patients entering ART with advanced HIV disease has remained relatively unchanged. In 2016, we estimated that of 654 868 patients entering care, 32.9% had advanced HIV disease, and 16.8% had very advanced HIV disease. Men were almost twice as likely as women (23.1% vs 12.6% ) to enter care with very advanced HIV disease.

**Conclusions:**

The proportion of patients presenting with advanced HIV disease in South Africa remains consistently high despite ART scale-up, representing a large and avoidable burden of morbidity. Early HIV diagnosis, rapid linkage to ART and approaches to attract men into early ART initiation should be prioritized.

The South African national HIV program has achieved substantial antiretroviral therapy (ART) coverage over the last decade, reaching 56% of all human immunodeficiency virus (HIV)–infected people in 2016, with 3.8 million adults on treatment [1]. Coverage expansion has been supported by a national strategic plan for prevention, treatment, care, support, and surveillance linked with policy [2] and guideline changes allowing for earlier ART initiation [3–6]. As ART scale-up continues, it is anticipated that increased access to treatment and reduced stigma will lead to treatment initiation earlier in HIV infection. Numerous studies have shown, both in South Africa [7] and elsewhere [8–10], that those who initiate ART at higher CD4 cell counts can expect a near-normal life expectancy and are at dramatically reduced risk of onward HIV transmission [11]. In contrast, a CD4 count <200 cells/µL is a strong predictor of severe morbidity, comorbidities such as tuberculosis and cryptococcal meningitis, and mortality [12, 13]. Guidelines for managing advanced HIV disease and rapid initiation of ART have been published recently [14].

Despite efforts to promote earlier treatment initiation, limited data are available on the existing burden of advanced HIV disease in South Africa and trends over time. Recent analysis of unlinked laboratory CD4 results suggests that the proportion of patients with advanced (CD4 <200 cells/µL) and very advanced (CD4 <100 cells/µL) HIV disease remains high [15], despite advances in access to and increased enrollment into care in recent years. Furthermore, an average of 10% of annual CD4 samples tested in the South African National Health Laboratory Service (NHLS) have a CD4 count <100 cells/µL, with a corresponding reported national positivity rate for cryptococcal antigenemia of 4%–5% [16, 17]. Because these CD4 samples are obtained from patients both prior to and after ART initiation, and because patients may have multiple records, it is difficult to make inferences from these unlinked CD4 data about the prevalence of advanced HIV among patients seeking care.

The aim of this study was to assess the distribution of ART-naive patients presenting with advanced and very advanced HIV disease, as indicated by a CD4 count <200 cells/µL and <100 cells/µL, respectively, and identify changes in distribution patterns over the last 10 years of scale-up of care.

## METHODS

The NHLS, being the sole provider of CD4 testing for the South African public sector since the onset of the HIV program, performed >3 million tests per year through a network of between 50 and 60 CD4 testing laboratories. These CD4 laboratory data can provide insights into the immunological status of patients in HIV care and can be used to identify trends and changes in the prevalence of advanced HIV disease over time. Historically, in the absence of a unique patient identifier, specimen-based laboratory data has been largely unable to differentiate between CD4 testing among those starting ART and those already on ART and being monitored during follow-up. We linked CD4 count and viral load monitoring tests taken from 1 April 2004 to 31 December 2016 using probabilistic linking methods adapted from the Fellegi-Sunter method [18, 19], based upon patient characteristics: name, surname, birth date, gender, facility, and province. We calculated a similarity score for each test as a weighted average over component scores, with weights optimized using manually matched training data taking into account the response frequency (rare elements are weighted more than common ones), adjusting for spelling differences in names using the Jaro-Winkler approach [18, 20], and inversions in names, and date elements. Compared against a manually matched gold standard, we found a sensitivity of 91.0% (ie, 9.0% undermatching) and positive predictive value of 90.5% (ie, 9.5% overmatching). Our primary outcome was entry into HIV clinical care as defined by a patient’s first CD4 count. To ensure that the CD4 tests included in the analysis were taken prior to ART initiation, we excluded patients who had either (1) a viral load test earlier than 30 days before the first CD4 test regardless of the viral load result or (2) a suppressed viral load result (<1000 copies/mL) within 30 days prior or 60 days after the first CD4 count test.

We included all patients aged ≥15 years as CD4 tests were less consistently used to determine treatment eligibility in the pediatric population and were considered to be a poor indicator of entry into care. We only included patients from the province of KwaZulu-Natal (KZN) from 1 July 2011 onward as the province did not start contributing tests to the NHLS until the second quarter of 2010.

We estimated numbers and proportions of patients with first CD4 counts of <100 cells/µL and 100–199 cells/µL for 12-month periods from 1 January 2005 to 31 December 2016. We calculated relative risk and 95% confidence intervals (CIs) to show differences in the proportion of patients presenting with advanced disease by sex. All analysis was conducted using SAS version 9.4 software (SAS Institute, Cary, North Carolina).

## RESULTS

### People Living With HIV Entering Care From 2005 to 2016

The probabilistic linking method yielded an estimated total number of adults (males and females >15 years) with a first CD4 test to be 8036919 (Table 1). The number of patients with a first CD4 increased steadily from 344189 in 2005 to a peak of 903118 in 2012 and then declining to 654868 in 2016. Of the total number of patients entering care from 2005 to 2016, 65% were females.

**Table 1.** Adult Females, Adult Males, and All Adults With First CD4 Count Test <100 or 100–199 Cells/µL, by Sex and Calendar Year of Test, South Africa

Year	Females			Males			Total			RR (95% CI) of Males With First CD4 Count 0–199 Cells/µL Compared to Females
	0–99 Cells/µL	100–199 Cells/µL	Females With First CD4 Test, No.	0–99 Cells/µL	100–199 Cells/µL	Males With First CD4 Test, No.	0–99 Cells/µL	100–199 Cells/µL	Persons With First CD4 Test, No.	
2005	24.4% (56409)	18.4% (42395)	230985	34.3% (38850)	20.0% (22597)	113204	27.7% (95259)	18.9% (64992)	344189	1.27 (1.26–1.28)
2006	22.6% (71024)	18.0% (56714)	314713	33.7% (48608)	20.0% (28849)	144110	26.1% (119633)	18.6% (85564)	458823	1.32 (1.32–1.33)
2007	20.9% (74618)	17.4% (61978)	356259	32.9% (52720)	19.9% (32011)	160471	24.6% (127338)	18.2% (93989)	516730	1.38 (1.37–1.39)
2008	19.7% (84757)	17.7% (76224)	429455	32.3% (62948)	20.6% (40167)	194682	23.7% (147706)	18.6% (116391)	624137	1.41 (1.41–1.42)
2009	17.3% (74291)	17.0% (72826)	428413	29.7% (59743)	20.4% (41065)	201298	21.3% (134034)	18.1% (113891)	629711	1.46 (1.45–1.47)
2010	16.2% (82834)	17.2% (88324)	512650	27.7% (69041)	20.7% (51623)	249265	19.9% (151875)	18.4% (139947)	761915	1.45 (1.44–1.46)
2011	14.4% (84913)	16.1% (95158)	590823	25.2% (78334)	20.2% (62930)	310888	18.1% (163248)	17.5% (158088)	901711	1.49 (1.48–1.50)
2012	14.0% (81201)	15.3% (89138)	581462	24.9% (80096)	19.9% (63965)	321656	17.9% (161297)	17.0% (153103)	903118	1.53 (1.52–1.54)
2013	13.0% (65050)	14.1% (70383)	498888	23.9% (69523)	19.1% (55601)	291487	17.0% (134573)	15.9% (125984)	790375	1.58 (1.57–1.59)
2014	12.8% (58940)	13.8% (63329)	459105	23.6% (66748)	18.9% (53544)	282794	16.9% (125688)	15.8% (116873)	741899	1.60 (1.59–1.61)
2015	12.4% (53728)	13.6% (59006)	432661	22.8% (63104)	19.0% (52561)	276782	16.5% (116831)	15.7% (111567)	709443	1.60 (1.59–1.62)
2016	12.6% (49603)	13.9% (54666)	392770	23.1% (60421)	19.3% (50472)	262098	16.8% (110024)	16.1% (105138)	654868	1.59 (1.58–1.61)
All years	16.0% (837369)	15.9% (830142)	5228184	26.7% (750137)	19.8% (555384)	2808735	19.8% (1587506)	17.2% (1385526)	8036919	

Data are presented as % (No.) unless otherwise indicated.

Abbreviations: CI, confidence interval; RR, relative risk.

### Proportion of Adults Entering Care With Advanced Disease

**Figure 1.**
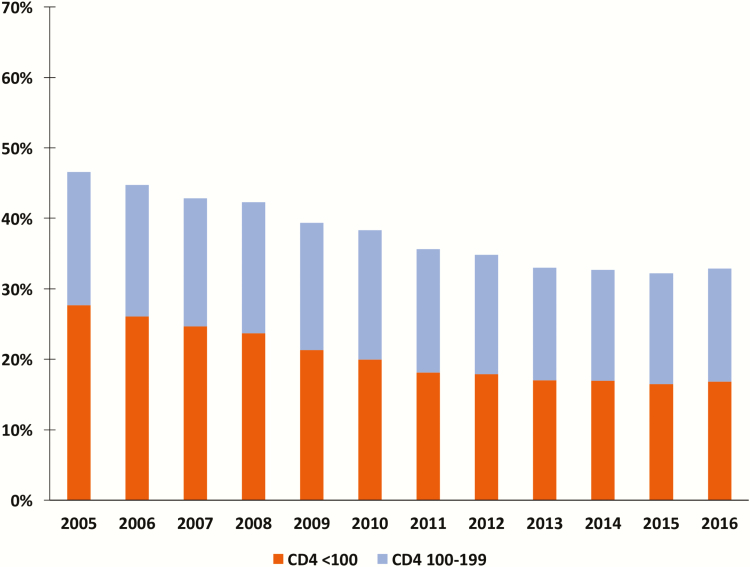
Proportion of patients entering care with advanced and very advanced HIV disease (first CD4 count test <100 and 100–199 cells/µL).

From 2005 to 2016, the proportion of patients with a CD4 count of <200 cells/µL entering into care declined from 46.6% to 32.9% (Figure 1). However, from 2012 to 2016 the proportion of patients presenting with advanced HIV disease remained relatively unchanged and ranged from 34.8% to 32.9%. The proportion of patients presenting with very advanced HIV disease (CD4 <100 cells/µL) fell from 27.7% in 2005 to 18.1% in 2011, but has remained unchanged over the last 5 years, at 16.8% in 2016.

### Proportion of Advanced HIV Disease Among Males and Females

**Figure 2.**
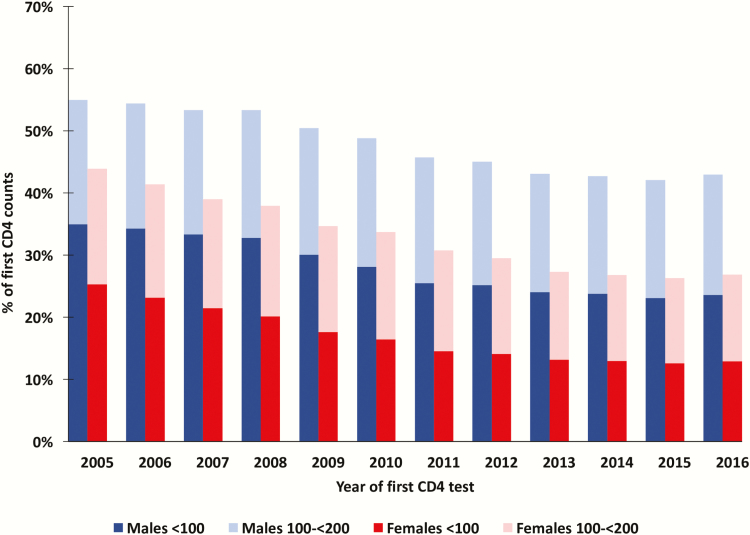
Proportion of males and females presenting to care with advanced and very advanced HIV disease from 2005 to 2016 (CD4 values presented as cells/µL).

**Figure 3.**
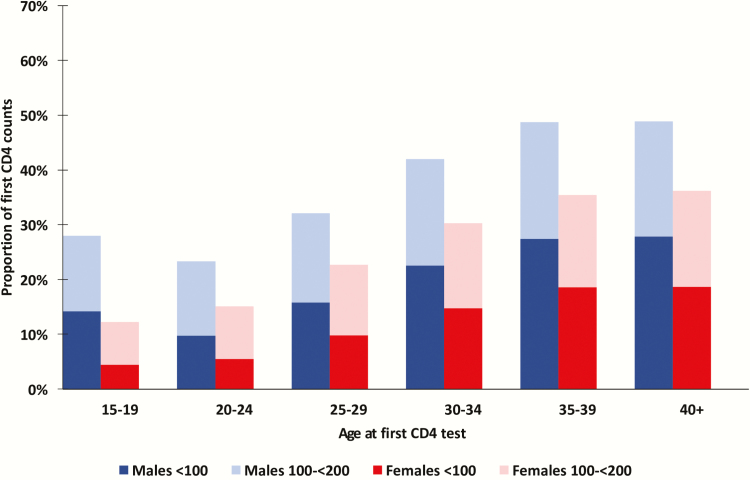
Distribution by age (in years) and sex of the proportions of first CD4 count test <100 and 100–199 cells/µL in 2016.

Between 2005 and 2016, the proportion of males presenting with advanced disease was consistently higher than the proportion of females presenting with advanced disease. However, the share presenting with advanced disease also fell faster for females over this period. Among males seeking care, the share with CD4 <200 cells/µL fell just 11 percentage points, from 54.3% in 2005 to 42.3% in 2016. In contrast, among women seeking care, the share with CD4 <200 cells/µL fell by 16 percentage points, from 42.8% in 2005 to 26.5% by 2016 (Figure 2), the male–female gap widening by nearly 50%. From 2012 to 2016, the proportion of all males with a first CD4 <100 cells/µL was close to double that among females with CD4 <100 cells/µL (Figure 2). The relative risk of males presenting with a first CD4 count of <100 cells/µL was 1.27 (95% CI, 1.26–1.28) in 2005 and has increased to 1.59 (95% CI, 1.58–1.61) in 2016 (Table 1). The excess burden of advanced and very advanced disease among males (vs females) was apparent in all age groups in 2016 (Figure 3).

## DISCUSSION

The number of people receiving ART through the South African national HIV program has increased 30-fold, from 103300 in 2005 to 3.8 million in 2016 [1]. Since the inception of the program, CD4 counts have been provided nationally, initially to guide treatment initiation decisions, as per World Health Organization guideline thresholds, and more recently to identify those with advanced HIV disease and therefore at higher risk for morbidity, mortality, and with greater medical need.

We found that while the proportion of patients presenting with advanced HIV disease has declined over the 12 years, it has remained unchanged over the last 5 years, with more than a third of patients entering care with a CD4 cell count <200 cells/µL. The absence of clinical symptoms suggestive of deteriorating health and the lack of incentives to be tested in an environment where stigma associated with HIV infection is still prevalent have contributed to maintaining the status quo. The persistently high burden of patients presenting with advanced HIV disease represents a substantial and avoidable burden of HIV morbidity, increased medical costs, and increased risk of onward transmission.

Our work is consistent with data from other reports from both high-income settings [21] and low- and middle-income settings, elsewhere [21–23] and in South Africa [15], which reported a general increase in average CD4 cell count at presentation and at initiation of ART. These gains are due in part to changes in policies supporting earlier access to treatment and improvements in drug efficacy and tolerability as well as formulation. Nevertheless, despite these gains, there is a persistent burden of patients who present late for care with advanced HIV disease. Furthermore, the sex disparities consistently increased over the study period, with the relative risk of males entering into care with advanced disease increasing in the later years. This concurs with the findings from 4 sub-Saharan countries, where the odds of advanced HIV disease at ART initiation were also higher among men and increased over time [24]. There are several factors that have resulted in these differences. The rapid ART scale-up has focused on women and children, supported by high-level political commitment [25] and funding [26]. Consequently, more women and children have accessed care and treatment and have done so earlier than men, who only contributed to 35% of those enrolled in ART programs [27]. This is further augmented by poor health-seeking behavior of HIV-positive men [28]. Therefore, ART programs will need to continue identifying barriers to access care and devise strategies to overcome them, as well as to ensure equitable access for both men and women.

These findings highlight the need to focus on access to rapid diagnosis and linkage to care, particularly among men, to support early identification and accelerated ART initiation. Nevertheless, it can be expected that even with continued improvements in early access to testing and treatment, a high proportion of patients will continue to present with advanced HIV disease. In addition, there is some evidence of an increasing number of patients presenting with advanced HIV disease following initiation and subsequent interruption of ART [29]. For such patients, the ability to identify advanced HIV disease—and therefore, vulnerability to severe morbidity and mortality—is critical to management of their infection and its sequelae. In the age of “Treat all,” CD4 testing remains essential to identify patients presenting with advanced disease and to tailor care to their needs.

The study has several strengths and limitations. The analysis presented here is based on a large nationally representative database from patients accessing the public-sector healthcare services and spans >12 years. The NHLS database does not include patients who seek access to clinical care in the private sector or some nongovernmental organizations; care utilization outside the public sector was estimated to be 8.5% in 2015 [30]. ART monitoring laboratories from KZN were not initially part of the NHLS network of laboratories, and patients from KZN were not included in our analysis until Q2 2011. Therefore, the results prior to that date do not represent the entire national population. In the absence of a unique patient identifier in South Africa, laboratory tests have been probabilistically linked with acceptable level of sensitivity and specificity. Still, linkage errors may have occurred, which could lead us to over- or underestimate the numbers of patients seeking care. We note that our estimate of >8 million patients includes all patients who presented to the public-sector HIV program and had blood drawn for a CD4 count, regardless of whether they were linked to further care and treatment. Our data also include patients who sought care in the early years of the program and may no longer be alive. We acknowledge that some contribution to the sex disparities likely originated from pregnant females, who have had additional opportunities to access care and be offered early HIV testing. However, estimates from a multicenter cohort study of adult patients initiating ART in South Africa reported only 7% of females to be pregnant [31]. Correction for this source of bias was not possible because the pregnancy status is not available in the NHLS database. Finally, our analysis was limited to first time care-seekers and did not assess the burden of advanced HIV disease among patients who have started ART, disengaged from care, and subsequently returned to care when they are sick. Therefore, our results are likely partial estimates of the total burden of advanced HIV disease in South Africa.

In conclusion, these findings show an enduring and substantial burden of advanced HIV disease, despite a period of intense scale-up of ART access. In addition to promoting earlier care-seeking, there is a continued need to be able to identify patients with advanced HIV disease and to target this population with an appropriate package of care and services to address the high risk of morbidity and mortality they face, as recently recommended by the World Health Organization.

## Notes

***Author contributions*.** S. C., J. B., C. N., B. M.-B., M. M., M. P. F., D. K. G., N. F., and W. M. designed the study; S. C., J. B., C. N., B. M.-B., M. M., M. P. F., N. F., and W. M. collected and analyzed the data; S. C., D. K. G., and N. F. wrote the first draft.

***Acknowledgments.*** The authors would like to acknowledge the assistance of Sue Candy and her team at the NHLS Corporate Data Warehouse in providing us with laboratory results data that made this research possible.

***Financial support.*** This work was made possible by the generous support of the American people through cooperative agreements AID 674-A-12-00029 (to M. M., J. B., M. P. F., C. N.) and AID- 674-A-12-0020 (to W. M., J. B.) from USAID. Research reported in this publication was supported by the National Institute of Allergy and Infectious Diseases (award number R01AI115979 to M. M., M. P. F., C. N.) and National Institute of Mental Health (award number 1K01MH105320-01A1 to J. B.), all of the NIH. J. B. receives support from the Boston University Peter Paul Career Development Professorship. This supplement was supported by funds from the Bill & Melinda Gates Foundation.

***Supplement sponsorship***. This article appears as part of the supplement “Advanced HIV Disease,” sponsored by the World Health Organization.

***Potential conflicts of interest.*** All authors: No reported conflicts of interest. All authors have submitted the ICMJE Form for Disclosure of Potential Conflicts of Interest. Conflicts that the editors consider relevant to the content of the manuscript have been disclosed.
